# Motion Analysis of Live Objects by Super-Resolution Fluorescence Microscopy

**DOI:** 10.1155/2012/859398

**Published:** 2011-11-17

**Authors:** Chunyan Yao, Jianwei Zhang, Guang Wu, Houxiang Zhang

**Affiliations:** ^1^Key Laboratory of E&M, Zhejiang University of Technology, Ministry of Education and Zhejiang Province, Hangzhou 310014, China; ^2^Department of Informatics, University of Hamburg, 22527 Hamburg, Germany; ^3^Guangxi Academy of Sciences, 98 Daling Road, Nanning 530007, China; ^4^DreamSciTech Consulting, Shenzhen 518054, China; ^5^Department of Technology and Nautical Sciences, Aalesund University College, 26025 Aalesund, Norway

## Abstract

Motion analysis plays an important role in studing activities or behaviors of live objects in medicine, biotechnology, chemistry, physics, spectroscopy, nanotechnology, enzymology, and biological engineering. This paper briefly reviews the developments in this area mostly in the recent three years, especially for cellular analysis in fluorescence microscopy. The topic has received much attention with the increasing demands in biomedical applications. The tasks of motion analysis include detection and tracking of objects, as well as analysis of motion behavior, living activity, events, motion statistics, and so forth. In the last decades, hundreds of papers have been published in this research topic. They cover a wide area, such as investigation of cell, cancer, virus, sperm, microbe, karyogram, and so forth. These contributions are summarized in this review. Developed methods and practical examples are also introduced. The review is useful to people in the related field for easy referral of the state of the art.

## 1. Background

Motion analysis for microscopic live objects has become a new approach to understanding complex living information of the microworld [1]. Fluorescence microscopy imaging has rapidly evolved in these years and provided new means for studying microbial behavior, cell motion, or intracellular processes in vivo. The super-resolution techniques allow the observation of many biological structures not resolvable in conventional fluorescence microscopy. New advances in these techniques now give them the ability to image three-dimensional (3D) structures, measure interactions, and record dynamic processes in living cells [2–5]. However, since such studies often generate a large volume of noisy image data that cannot be analyzed efficiently and reliably by human observation, it is a critical issue to develop computing methods for automatic motion analysis. Many tracking techniques exist in the computing science but often fail to yield satisfactory results in the case of high object densities, high noise levels, and complex motion patterns [6].

Cellular life can be described as a dynamic equilibrium of a highly complex network of interacting molecules [7]. It is not sufficient to only know the identity of the participants in a cellular process, but questions such as where, when, and for how long also have to be addressed to understand the mechanism being investigated. 

Recent improvements in methods of single-particle fluorescence tracking have permitted detailed studies of molecular motion on the nanometer scale due to its high selectivity, sensitivity, simplicity, and fastness [8]. The high microscopy sensitivity resolves the signal of a single fluorescence-labeled bimolecular within a living cell. A time resolution of milliseconds for imaging weakly fluorescent cellular structures like small organelles, vesicles, or even single molecules is already available [9]. This introduces tools to a burgeoning field of nanotechnology for analysis of microtubules, DNAs, proteins, and other biochemical particles.

Motion analysis for microscopic live objects is a key issue in this field. One aim is the recognition of selected targets in the image and tracking them in time [10]. Single-particle tracking is powerful in measuring particle motion from video-microscopy image sequences [11, 12]. A system actually needs to observe the type of cells and their movement in long image sequences [13].

In the survey, advances in motion analysis for microscopic live objects are briefly reviewed. Overall, significant progress has been made in several issues, as well as being integrated with other methods for biomedical applications.

The scope of this paper is restricted to motion analysis for microscopic live objects by image processing [14, 15], especially in the field of cellular research. Although this topic has attracted researchers as early as since the 1980s, this survey concentrates on the contributions of the latest three years when the single-molecule fluorescence microscopy or super-resolution fluorescence imaging became practically available. It includes only some representative or important works from recent years.

The current paper has four more sections.  Section 2 introduces some application examples.  Section 3 briefly shows the principle of particle filters.  Section 4 lists the relevant research problems and typical methods.  Section 5 includes a conclusion and a discussion of future trends.

## 2. Application Examples

### 2.1. Virion Study

Optical studies have revealed that virions move laterally on the plasma membrane, but the complexity of the cellular environment has prevented access to the molecular dynamics of early virus-host couplings, which are important for cell infection [16, 17]. Kukura et al. discussed a technique for studying virus-membrane interactions and for resolving nanoscopic dynamics of individual biological nanoobjects [18]. Helmuth et al. uses a shape reconstruction method that leads to better discrimination between different endosomal virus entry pathways and to more robust, accurate, and self-consistent quantification of endosome shape features [19].

For 3D tracking of GLUT4 vesicles, Wu et al. present an algorithm, where mobile granules are segmented from a time-lapse image stack, and Kalman filter is used to estimate the granules for reducing searching range of reliable tracking [20]. Schelhaas et al. studied the lateral mobility of individual, incoming human papillomavirus type 16 pseudoviruses bound to live HeLa cells by single-particle tracking [21, 22].

In [23], eight tracking approaches are evaluated for testing real microscopy images of HIV-1 particles. The probabilistic approaches based on independent particle filters are found to be superior to the deterministic schemes as well as to the approaches based on a mixture of particle filters.

### 2.2. Cellular Analysis

A tracking algorithm is tested in [10] on solid-lipid nanoparticles diffusing within cells and on lymphocytes diffusing in lymphonodes. It appears useful for the cellular and in vivo image processing where little a priori assumption on the type, the extent and the variability of particle motions, can be done. Another example of single-particle tracking is proposed in [13] with application to platelet adhesion under flow.

### 2.3. Subcellular Analysis

Determination of the subcellular localization and dynamics is an important step towards the understanding of multimolecular complexes in a cellular context [24]. Live imaging of subcellular structures is essential for the understanding of cellular processes. Algorithms have been proposed in the community for detecting fluorescently labeled subcellular objects in microscope images [25]. However, reconstruction of subcellular structures from images remains a major challenge in the field [19].

Transport of intracellular organelles along the microtubule cytoskeleton is in a bidirectional manner [26]. The study of protein dynamics is essential for the understanding the multi-molecular complexes at subcellular levels [27]. For understanding of microtubules and their associated proteins, dynamic interactions are routinely observed in vitro on the level of single molecules, mainly using a geometry in which labeled motors move on surface-immobilized microtubules [28]. Machan and Hof discussed several key questions of lateral mobility investigation in planar lipid membranes, including the influence of membrane and aqueous phase composition, choice of a fluorescent tracer molecule, frictional coupling between the two membrane leaflets and between membrane and solid support [29]. A simulation and estimation framework is proposed in image-processing field for intracellular dynamics and trafficking in video-microscopy and fluorescence imagery [30].

### 2.4. Activity in Nucleus

The nucleus is a well-organized and highly compartmentalized organelle, and this organization is intimately related to nuclear function [31]. Dange et al. discussed the potential of tracking single RNAs and proteins in the nucleus [7]. The dynamics, localization, and interaction rates are vital to the understanding of cellular life. They provide a review of the HIV life cycle, which provides an opportunity to study mechanisms deeply integrated within the structure of the nucleus.

Gene delivery helps treatment of many diseases. Underlying that the rational design of gene-delivery vectors can discover the individual steps of the gene-delivery pathway. With fluorescence microscopy, it is possible to isolate individual steps along the gene-delivery pathway to characterize the mechanisms of cellular binding, cellular internalization, and nuclear entry [32].

3D imaging of bacterial protein distribution and neuron dendritic morphology is discussed in [33]. Super-resolution method is proposed in [34] for orientation estimation and localization of fluorescent dipoles using 3D steerable filters.

### 2.5. Molecular Mechanism

Dynamic properties of proteins have crucial roles in understanding protein function and molecular mechanism within cells [35]. Researchers often need to accurately determine the motion of single-molecule trajectories [36]. It is also interesting to study the behavior of motor proteins and associated organelle transport within a cell [11, 37]. Molecular motors such as kinesin, myosin, and F-1-ATPase are responsible for many important cellular processes. These motor proteins exhibit nanometer-scale, stepwise movements on millisecond timescales. Methods to measure these small and fast movements with high spatial and temporal resolution require relatively complicated systems [38]. Colocalization of two or more molecules is an essential aspect of many biological molecular processes and single-molecule technologies for investigating these processes in live cells [39]. An imaging method is developed in [40] to quantitatively detect the colocalization of two species of individual molecules.

## 3. Particle Tracking

### 3.1. Particle Filtering

Probabilistic tracking methods have recently shown several advantages with better integration of spatial and temporal information, and the possibility to more effectively incorporate prior knowledge. Smal et al. propose a fully automated particle filtering algorithm for the tracking of many subresolution objects in fluorescence microscopy image sequences [6]. It involves a track management procedure and allows the use of multiple dynamics models. 

Villa et al. developed a particle-tracking algorithm optimized for low signal noised images with minimum requirements on the target size and without a priori knowledge of the motion type. The particle tracking is performed by building, from a stack of accumulative difference images, a single 2D image in which the motion of the whole set of the particles is coded in time [10].

Many single-particle tracking algorithms deliver subpixel accurate measurements with noisy data corresponding to sub-10-nm resolution. Image-correlation techniques have been shown to be the most accurate method of tracking extended objects. Saunter proposes a method for experimentally determining the accuracy of image-correlation-based tracking and demonstrates the possibility of making measurements accurate to 5 nm when working with extended objects within live cells [11].

### 3.2. Multiple Model Filtering

A multiple model tracking method is proposed in [41] with multidimensional assignment. They combine an interacting multiple model filter, multidimensional assignment, particle occlusion handling, and merge-split event detection together. The advantage of a multidimensional assignment is that both spatial and temporal information can be used by using several later frames as reference. The multiple model filter, which is used to maintain and predict the state of each track, contains several models which correspond to different types of biologically realistic movements. 

Godinez et al. developed deterministic and probabilistic approaches for multiple virus tracking in multichannel fluorescence microscopy images [23]. The probabilistic approaches are based on a mixture of particle filters and independent particle filters.

Genovesio et al. propose a method to detect and track multiple moving biological spotlike particles showing different kinds of dynamics [42]. It can extract and analyze information such as number, position, speed, movement, and diffusion phases of particles. After a detection stage performed by a 3D undecimated wavelet transform, prediction of spots' future states is accomplished with an interacting multiple model algorithm which includes several models corresponding to different biologically realistic movement types. Then the filters are updated to compute final estimations.

For tracking problem of several hundreds of objects, a framework is provided in [43] with general information about vesicle transport, that is, traffic flows between origin and destination regions. Traffic estimation is accomplished by adapting the advances in network tomography. A method is proposed in [44] to detect and track multiple moving biological spotlike particles showing different kinds of dynamics in image sequences.

### 3.3. 3D Nanoscopy

Various biophysical studies require high spatial and temporal resolution in vitro and also in vivo [38]. It remains a challenge to precisely localize single molecules in 3D [45, 46]. Tang et al. applies near-isotropic 3D optical nanoscopy with photon-limited chromophores to 3D imaging of bacterial protein distribution and neuron dendritic morphology with subdiffraction resolution [3, 33].

Huang et al. demonstrated 3D stochastic optical reconstruction microscopy by using optical astigmatism to determine both axial and lateral positions of individual fluorophores with nanometer accuracy [2]. The construction of a 3D image can achieve a resolution of 20 to 30 nanometers in the lateral dimensions and 50 to 60 nanometers in the axial dimension. This allows to resolve the 3D morphology of nanoscopic cellular structures.

Yu et al. argued that the bright fluorescence can yield a theoretical particle tracking uncertainty of less than 1 nm. A lateral tracking uncertainty of 1-2 nm is determined from analysis of trajectories of fixed and freely diffusing particles. Axial position information for 3D particle tracking is obtained by defocused imaging [47].

## 4. Research Issues

### 4.1. Fluorescence Microscopy

Most biological molecules are less than 5–10 nm in diameter, and getting molecular details requires imaging at this scale. The basic research in cell biology and in medical sciences is mainly based on confocal fluorescence [10]. Nowadays, a great deal of attention in biomedical and pharmaceutical technology is going to the development of nanoscopic particles to efficiently deliver nucleic acids to target cells. Despite the great potential of nucleic acids for treatment of various diseases, progress in the field is fairly slow [48]. The resolution of conventional optical microscopy is constrained to about 200–500 nm due to the diffraction limit, but the recent developed super-resolution fluorescence imaging, which is based on single-molecule localization and image reconstruction, offers a comparatively simple way to achieve a substantially improved optical resolution down to similar to 20–50 nm in the image plane [49–51]. The recent stochastic optical reconstruction microscopy makes use of single-molecule imaging methods and photo-switchable fluorescent probes to temporally separate the spatially overlapping images of individual molecules. An image is acquired over a number of imaging cycles. This allows the position to be determined with nanometer accuracy [51]. Single-molecule fluorescence microscopy has become one of the most popular methods in the single-molecule toolbox [52]. In [53], a 26-ms time resolution and a spatial accuracy of 5 nm in each dimension are achieved. The resulting high-resolution trajectories reveal not only heterogeneity among vesicles but also heterogeneity within single-vesicle trajectories. Fluorescence imaging with one-nanometer accuracy and single-molecule high-resolution colocalization are used to monitor the diffusive behavior of synthetic molecular walkers at the single-molecule level. Michelotti discussed the imaging methods and experimental challenges of very low velocities (e.g., 3 nm/min) of nanowalkers [8]. The state of the art in single-molecule tools including fluorescence spectroscopy, tethered particle microscopy, optical and magnetic tweezers, and atomic force microscopy can be found in the survey by Walter et al. [54].

### 4.2. Detection

Determination of the position of a cell, termed localization, is of paramount importance in achieving reliable and robust motion analysis. Achieving high-level descriptors such as dynamics and activities is possible if the position is known and accurately tracked. Aguet et al. introduce a method for the joint estimation of position and orientation of dipoles, based on the representation of a physically realistic image formation model as a 3D steerable filter. They establish theoretical, localization-based resolution limits on estimation accuracy and experimentally show that the position accuracy is 5 nm and the orientation accuracy is 2 degrees [34].

Smal et al. evaluate the performance of the most frequently used detection methods for quantitative comparison [55]. Seven unsupervised and two supervised methods are involved. The experiments are carried out on synthetic images of three different types, for which the ground truth was available, as well as on real image data sets acquired for two different biological studies. The results suggest that for very high noise images the supervised learning methods perform best overall.

### 4.3. Intracellular Process

Ensemble measurements are not sufficient to describe individual steps of molecular mobility, spatial-temporal resolution, kinetic parameters, and geographical mapping. It is important to find where individual steps exactly occur for better understanding of the living cell. The nucleus is with many highly complex multiorder processes, such as replication, transcription, splicing, and so forth and provides a complicated, heterogeneous landscape. Single-molecule tracking has become more and more attractive [7]. Jung et al. reported the work on diffusion of oriented single molecules with switchable mobility in networks of long unidimensional nanochannels [56].

Using dynamic techniques to track, manipulate, and probe motor proteins is crucial in providing new insights [1]. To understand the regulation of intracellular transport through quantitative analysis of the motion of organelles in a controlled environment, Soppina et al. present a simple and reliable method that uses avidin-coated magnetic beads to prepare microtubules labeled at the minus end. It demonstrated video-rate high-resolution imaging of single cellular organelles moving along plus and minus directions on labeled microtubules [26].

### 4.4. Segmentation and Count

Ruusuvuori carried out a comparative study including eleven-spot detection or segmentation algorithms from various application fields [25]. The experimentally derived images permit a comparison of method performance in realistic situations where the number of objects varies within image set. The study finds major differences in the performance of different algorithms, in terms of object counts and segmentation accuracies. An automatic segmentation and tracking method is designed in [44] to enable quantitative analyses of cellular shape and motion from 4D microscopy data.

A method in [13] includes functions of automatic segmentation methodology which removes operator bias, platelet recognition across the series of images based on a probability density function. It can be integrated to analyze the platelet trajectories to obtain relevant information, such as deposition and removal rates, displacement distributions, pause times, and rolling velocities.

### 4.5. Movement and Tracking

It is often required to localize particles in a live cell to a certain accuracy to study their localization-related functions [57, 58]. Particle tracking has seen numerous applications in live-cell imaging studies of subcellular dynamics. Establishing correspondence between particles in a sequence of frames with high particle density, particles merging and splitting, particles entering and exiting the frame, temporary particle disappearance, and an ill-performing detection algorithm is the most challenging task [41]. Smal et al. propose a completely automatic tracker based on a Bayesian probabilistic framework. It better exploits spatiotemporal information and prior knowledge than common approaches, which yields more robust tracking [59].

Sbalzarini and Koumoutsakos present a 2D feature tracking algorithm for the automated detection and quantitative analysis of particle trajectories as recorded by video imaging in cell biology [60]. The tracking requires no a priori modeling of the motion, it is self-initializing, it discriminates spurious detections, and it can handle temporary occlusion as well as particle appearance and disappearance from the image.

To extract the maximum information from a sequence of fluorescence images, Yoon et al. describe a Bayesian-based inference approach, based on a transdimensional sequential Monte Carlo method that utilizes spatiotemporal information. The method allows accurate tracking of molecules over long trajectories even with low signal/noise ratio and in the presence of fluorescence blinking and photobleaching [61].

The movement trajectories in fluorescence video microscopy can be computationally analyzed in terms of diffusion rate and mode of motion [21]. The trajectories play a role in the analysis of living-cell dynamics. Boulanger et al. develop a general simulation framework to produce image sequences showing small moving spots in interaction and corresponding to intracellular dynamics and trafficking in biology [30]. Wieser and Schutz describe strategies how to make use of single-molecule trajectories for deducing information about nanoscopic structures in a live cell context, for example, elucidating the plasma membrane organization [9].

For simultaneously determining multiple trajectories of single molecules from sequential fluorescence images, Claytor et al. developed a procedure for accurately monitoring surface motion under ambient conditions [36]. The tracking algorithm is computationally nondemanding and does not assume a model for molecular motion.

### 4.6. Dynamics

Investigation of lipid lateral mobility in biological membranes and their artificial models provides information on membrane dynamics and structure where methods based on optical microscopy are convenient for such investigations [29].

The motion of the tagged locus is observed and analyzed to extract quantitative information regarding its dynamics. Levi and Gratton reviewed recent advances in chromatin dynamics in interphase. They introduced the basis of particle-tracking methods and trajectory analysis and summarized what has been learnt by using this technology in the context of chromatin dynamics [31]. Nitzsche reviewed the recent methods related to gliding motility assays in conjunction with 3D-nanometry [28]. They provide practical advice on how to set up gliding assays, acquire high-precision data from microtubules and attached quantum dots, and analyze data by 3D-nanometer tracking. Nanoscale tracking of single particles in fixed cells was demonstrated in [47], where a range of complex behaviors, possibly due to binding/unbinding dynamics, is observed. 

For direct visualization of the protein dynamics, Wang et al. combined total internal reflection fluorescence microscopy with oblique illumination fluorescence microscopy to observe the movement of membrane-anchored green fluorescence proteins in living cells [35]. 

Kukura et al. present a colocalization methodology that combines scattering interferometry and single-molecule fluorescence microscopy to visualize both position and orientation of single quantum dot-labeled Simian virus 40 particles [18]. Using nanometer spatial with 8 ms temporal resolution, they observed sliding and tumbling motions during rapid lateral diffusion on supported lipid bilayers and repeated back and forth rocking between nanoscopic regions separated by 9 nm.

### 4.7. Events and Behaviors

Detection of meaningful events in spatiotemporal fluorescence image sequences is certainly important in cellular analysis, for example, membrane transport and gene-delivery process [32]. Pécot et al. propose an original patch-based Markov modeling to detect spatial irregularities in fluorescence images with low false alarm rates [27]. Toprak and Selvin described some of the most commonly used fluorescence imaging tools to measure nanoscale movements and the rotational dynamics of biomolecules [62].

### 4.8. Reconstruction

Shape reconstruction of 2D/3D subcellular structures from live cell helps us to understand the whole object. Helmuth et al. presented model-based algorithm to reconstruct outlines of subcellular structures using a subpixel representation [19, 63]. From 2D images, 3D tracking can also be realized by utilizing the exponential decay of the fluorescence intensity excited by the evanescent field [64].

## 5. Conclusion and Future Directions

This paper summarizes recent developments in motion analysis for microscopic live objects in cellular research. Typical contributions are addressed for localization, tracking, count, estimation, reconstruction, modeling, cell analysis, and so forth. Representatives are listed for people to have a general overview of state of the art. A number of methods are introduced on motion analysis for microscopic live objects. Most of the cited works are published reports in the last three years.

Although motion analysis for microscopic live objects has been developed in many applications as an important approach to observation and diagnosis, many problems still exist in its development for biomedical engineering. Researchers are exerting efforts not only in simple localization, but also in improving the future methods in other aspects.

1. Dealing with noisy data: in fluorescence microscopy, the blurred noisy images acquired are complex to analyze. Hidden Markov models facilitate extraction of the sequence of hidden states from noisy data through construction of probabilistic models. Since constraints resulting from short data sets and poisson-distributed photons in like fluorescence can limit good statistics, additional information criteria such as peak localization error and chi-square probabilities can incorporate theoretical constraints in principle [65].
2. Accurate detection: in live-cell fluorescence microscopy imaging, the biological images generally need to detect many subresolution objects. Indeed, complex interactions between a large number of small moving particles in a complex scene cannot be easily modeled, limiting the performance of object detection and tracking algorithms [30]. Object detection and tracking often perform poorly in the case of noisy image data [59]. In live-cell imaging by fluorescence microscopy, the signal-to-noise ratio can be extremely low, making automated spot detection a very challenging task [55].
3. Reliable tracking: reliability is a great concern in practical applications. Motion analysis relies on many conditions and parameters. Related techniques rely on existing noise statistics, initial positions, and sufficiently good approximation. Quantitative analysis of dynamic processes in living cells requires tracking of hundreds of bright spots in noisy image sequences [59]. Factors, such as low signal/noise ratio, unknown number of particles, and fluorophore blinking and photobleaching, make accurate tracking over long trajectories very difficult [61]. Existing tracking methods rarely work when many small and poorly distinguishable objects interact. Pécot et al. proposes that another way of tracking that consists in determining the full trajectories of all the objects, can be more relevant [24]. Techniques like background subtraction and Kalman filter are also helpful for reliable tracking [20]. Rogers et al. designed an algorithm to accurately track the motion of low-contrast particles against a background with large variations in light levels. The method is based on a polynomial fit of the intensity around each feature point and is especially suitable for tracking endogeneous particles in the cell, imaged with bright field, phase contrast or fluorescence optical microscopy. It can also simultaneously track particles of all different sizes and shapes [66].
4. Information analysis: advanced methods in computer vision and image processing are helpful to extract interesting information. Data analysis requires deep collaboration between biologists and computer scientists. It is a challenge to transform the vast amounts of unstructured data into quantitative information for the discovery of cellular behaviors and the rigorous testing of mechanistic hypotheses [67, 68].
